# Isolation and genotyping of viable *Toxoplasma gondii* from sheep and goats in Ethiopia destined for human consumption

**DOI:** 10.1186/1756-3305-7-425

**Published:** 2014-09-04

**Authors:** Endrias Zewdu Gebremedhin, Mukarim Abdurahaman, Tesfaye Sisay Tessema, Getachew Tilahun, Eric Cox, Bruno Goddeeris, Pierre Dorny, Stephane De Craeye, Marie-Laure Dardé, Daniel Ajzenberg

**Affiliations:** Faculty of Agriculture and Veterinary Sciences, Department of Veterinary Laboratory Technology, Ambo University, P. O. Box 19, Ambo, Ethiopia; College of Agriculture and Veterinary Medicine, Jimma University, P. O. Box 307, Jimma, Ethiopia; Institute of Biotechnology, College of Natural and Computational Sciences, Unit of Health Biotechnology, Addis Ababa University, P. O. Box. 1176, Addis Ababa, Ethiopia; Aklilu Lemma Institute of Pathobiology, Addis Ababa University, P. O. Box. 1176, Addis Ababa, Ethiopia; Faculty of Veterinary Medicine, Ghent University, Salisburylaan 133, Merelbeke, B-9820 Belgium; Department of Biosystems, Faculty of Bioscience Engineering, Catholic University of Leuven, Kasteelpark Arenberg 30 bus 2456, Heverlee, B-3001 Belgium; Department of Biomedical Sciences, Institute of Tropical Medicine, Antwerp, B2000 Belgium; Scientific Institute of Public Health, Communicable and Infectious Diseases, National Reference Center for Toxoplasmosis, Engelandstraat 642, Brussels, B1180 Belgium; Centre National de Référence (CNR) Toxoplasmose / Toxoplasma Biological Resource Center (BRC), Centre Hospitalier-Universitaire Dupuytren, Limoges, 87042 France; INSERM UMR 1094, Neuroépidémiologie Tropicale, Laboratoire de Parasitologie-Mycologie, Faculté de Médecine, Limoges, 87025 France

**Keywords:** *Toxoplasma gondii*, Bioassay, DAT, Ethiopia, Sheep, Goat, Genotyping, Microsatellite, Atypical, Virulent

## Abstract

**Background:**

*Toxoplasma gondii* is an obligate intracellular protozoan parasite that infects humans and a broad spectrum of warm-blooded vertebrates. The present study was undertaken with the objectives of isolation and determining the genotypes of *T. gondii* strains from sheep and goats slaughtered in East and West Shewa Zones of Oromia Regional State, Central Ethiopia.

**Methods:**

Hearts of 47 sheep and 44 goats that were seropositive in the Direct Agglutination Test (DAT) were bioassayed in mice. A multiplex PCR assay with 15 microsatellite markers was employed for genotyping of *T. gondii* isolates from sheep and goats.

**Results:**

Viable *T. gondii* were isolated from 47 (51.65%) animals, 27 sheep and 20 goats. Most isolates caused sub-clinical infections in mice, however, 2 sheep and 1 goat isolates were mouse-virulent, killing mice between 19–27 days post-inoculation. The success of *T. gondii* isolation in mice increased significantly (*P* = 0.0001) with higher DAT antibody titers in sheep and goats. Genotyping revealed that 29 (87.88%) of the 33 isolates were Type II, 3 (9.09%) were Type III and 1 (3.03%) was atypical. Three strains (one type II, one type III, and the atypical genotype) were virulent for mice.

**Conclusions:**

*T. gondii* tissue cysts in sheep and goats slaughtered for human consumption are widespread. This is the first report on isolation and genotyping of *T. gondii* from sheep and goats of Ethiopia.

**Electronic supplementary material:**

The online version of this article (doi:10.1186/1756-3305-7-425) contains supplementary material, which is available to authorized users.

## Background

*Toxoplasma gondii* is an obligate intracellular protozoan parasite that infects humans and a broad spectrum of warm-blooded vertebrates [1]. In humans, accidental ingestion of oocysts shed in feline faeces and consumption of raw or undercooked meat containing *T. gondii* tissue cysts are the major routes of transmission. In addition, if infection occurs during pregnancy, the parasite can cross the placenta and infect the fetus (congenital transmission) leading to abortion or congenital abnormalities [2, 3].

Previously, it was described that *T. gondii* has a clonal population structure consisting of three genetic lineages i.e., Type I, Type II and Type III [4]. However, recent studies with multilocus markers indicate greater genetic diversity than initially thought among isolates of *T. gondii* worldwide [5, 6]. *Toxoplasma gondii* presents a complex population structure with a mix of clonal and sexual propagation [5]. Epidemiological and population studies with multilocus PCR-RFLP or microsatellite markers have shown that *T. gondii* isolates from South America are highly diverse and distinct from those from North America and Europe, where Type II is predominant [5–13].

Few data are available on toxoplasmosis in both public health and veterinary sectors in Ethiopia [14]. Genotyping of *T. gondii* isolates using RFLP-PCR from 27 feral cats from Addis Ababa indicated limited genetic diversity [15].

The microsatellite (MS) genotyping technique is an easy-to-use and rapid genotyping method, which aims to ensure both levels (lineage and fingerprinting) of genetic discrimination of *T. gondii* isolates in a single multiplex PCR assay, using 15 MS markers located on 11 different chromosomes of *T. gondii* [9, 16]. The association of the 15 MS markers with different mutability patterns enables two different levels of genetic resolution for differentiating *T. gondii* strains at the typing level (types I, II, and III versus atypical strains) and at the fingerprinting level (closely related isolates within a clonal lineage). Microsatellite genotyping has a similar sensitivity with that of intron sequencing and a better resolution level to distinguish genetically closely related strains compared to PCR-RFLP [16].

The aim of the present study was to isolate and genotype *T. gondii* strains from sheep and goat heart tissues in Central Ethiopia.

## Methods

### Naturally exposed sheep and goats

Most of the sheep and goats studied originated from Adea, Fentale (East Shewa Zone) and Ambo (West Shewa Zone) districts. The location, altitude, climate, breed and production system of sheep and goats of these districts have been described previously [17].

Sheep and goats of the study areas slaughtered for human consumption at an export abattoir in Debre Zeit town were traced and identified using information supplied by the attendant and the abattoir manager. Production system, breed and purpose of keeping small ruminants (for meat and milk) are as described previously [17, 18]. In this study, sheep and goats aged over six months were sampled. The majority of the animals were males; few females were sampled because they are normally kept for breeding purpose and majority of those arriving at the abattoir are culls and infertile animals.

### Identification of seropositive animals

As part of an epidemiological study of *T. gondii* in sheep and goats of the East and West Shewa Zones of Central Ethiopia, 628 sera were tested using the direct agglutination test (DAT) (Toxo screen DA, Biomerieux®, France). The present study was undertaken from September, 2011 to June, 2013 using hearts of 91 DAT seropositive animals (47 sheep and 44 goats) and 14 seronegative animals (10 sheep and 4 goats) from previous seroepidemiological study [18].

### Bioassay of sheep and goats hearts for *T. gondii*

Approximately 50 g of heart tissue was prepared as described previously [1]. Briefly, each sample was cut in small pieces of approximately 1 cm^3^, homogenized in a blender for 30 sec, followed by suspension in 125 ml of saline solution (0.14 M NaCl) for another 30 sec. After homogenization, 250 ml of a pepsin solution (i.e. porcine stomach pepsin of 1:0000 biological activity 5.2 g, NaCl 10.0 g, HCl 14 ml, and distilled water to make 1,000 ml, pH 1.10–1.20) (Merck KG.A, Darmstadt, Germany) was added. After an incubation of 1 h at 37°C, the homogenate was filtered through two layers of gauze and centrifuged at 1200 × *g* for 10 min. The supernatant was discarded and the pellet was resuspended in 15 to 20 ml of 1.2% sodium bicarbonate solution (pH = 8.3) and re-centrifuged at 1200 × *g* for 10 min. The supernatant was discarded and the sediment was re-suspended in 5 to 10 ml of antibiotic saline solution (1000 U/ml penicillin and 100 μg streptomycin/ml in saline solution). One milliliter of this suspension was inoculated intraperitoneally (i.p.) in five mice per sample [1]. Bioassays were performed within 1 to 3 days after the slaughter of the animals. The mice used were *T. gondii* seronegative female Swiss Albino mice, obtained from the animal facility of the National Veterinary Institute, Debre Zeit, Ethiopia. Non-infected mice (n = 5) were kept separately as negative controls. The mice were given commercial pelleted feed and municipal chlorinated water ad libitum.

The inoculated mice were observed daily for the presence of clinical signs until day 60 post inoculation. The number of survivors, the presence of clinical signs, the weight and the eventual mortality (day of death) were recorded. A *T. gondii* isolate was considered virulent if mortality of mice was observed within four weeks of infection [19].

The mice were bled on day 60 post-inoculation and their sera tested for *T.gondii*-antibodies by the direct agglutination test (DAT) following the procedure described by manufacturer of the kit (Toxo screen DA, biomerieux®, France) as described previously. Sera were assayed at dilutions of 1/40 and 1/4000; a titer of 1: 40 was considered indicative of *T. gondii* exposure.

Two months after intraperitoneal (i.p) inoculation, the brain from surviving mice was removed after euthanasia with di-ethyl ether. Each brain was homogenized in 1 ml PBS (pH 7.2) using a mortar and pestle. Following microscopic examination and counting of cysts, the homogenates were stored in 1.5 ml Eppendorf tubes and frozen at −20°C until DNA extraction was done. The number of cysts in three aliquots of each 10 μl was counted under a light microscope with a 100X magnification. The total number of cysts in the brain of each mouse was determined by converting the sum of cysts in 30 μl to the whole volume of the brain homogenates [1, 20, 21]. A bioassay was considered positive if *T. gondii* cysts were detected in any of the five inoculated mice.

### DNA extraction

DNA was extracted from mice brain as described previously by Su and Dubey [22] using the QIAamp Tissue kit (Qiagen). Briefly, 75–100 μl of homogenized brain (approximately 25 mg brain tissue), 180 μl of lysis buffer of the kit (ATL) and 20 μl of proteinase K were added and incubated at 56°C until the tissue was completely lysed (60 to 90 min). The lysate was then mixed with 200 μl AL buffer and incubated for another 10 min at 70°C. DNA was precipitated by addition of 200 μl ethanol (96–100%). Then, the mixture was carefully applied to the QIAamp Mini spin column and centrifuged at 6000 × g for 1 min. The columns were then washed by centrifugation using buffers AW1 and AW2, according to the manufacturer’s instruction. Finally, the DNA was eluted from the column using 50 μl of the elution buffer of the kit (AE) and stored at −20°C until used for genotyping.

### Multiplex-PCR Microsatellite genotyping

*T. gondii* strains were genotyped using 15 microsatellite markers distributed on 11 of 14 chromosomes in a single multiplex PCR-assay, as described previously [16] (see Additional file 1 for details). Briefly, for each primer pair, the forward primer was 5’-end labeled with fluorescein to allow sizing of PCR products that were separated by electrophoresis in an automatic sequencer. PCR was carried out in a 25 μL reaction mixture consisting of 12.5 μl multiplex PCR Master Mix (Qiagen, France), 7.5 μl primer mix for 15 MS, and 5 μl of DNA template. Cycling conditions were 15 min at 95°C; 30 s at 94°C, 3 min at 61°C, and 30 s at 72°C (35 cycles); and the last extension step was 30 min at 60°C. PCR products were diluted 1/10 in deionized formamide. One μl of each diluted PCR product was further diluted 1/25 in 23.5 μl of deionized formamide and 0.5 μl of a dye-labeled size standard (ROX 500; Applied Biosystems). This mixture was denatured at 100°C for 5 min and then electrophoresed using an automatic sequencer (ABI PRISM 3130xl; Applied Biosystems). The sizes of the alleles in base pairs were estimated using GeneMapper analysis software (version 4.0; Applied Biosystems).

### Data analysis

Data generated were recorded and coded using Microsoft Excel and analyzed using STATA version 11.0 for Windows (Stata Corp. College Station, TX, USA). Descriptive statistics such as, percentage, mean, variance, etc. were used to summarize the data. The rate of cyst isolation in mice was compared with the DAT end titer of sheep and goats using the Chi-squared test. The Mann–Whitney U test was used to assess the relationship between parasite isolation in mice and *T. gondii* antibody end titer of sheep and goats, *i.e.,* the median sheep and goats antibody titers related to isolation and no isolation. Fisher’s Exact test was used to compare associations between *T. gondii* genotypes and species (sheep, goat), origin (West Shewa, East Shewa), severity (none, moderate, high), number of cysts (≤200, 201–500, ≥ 501) and DAT titer (≤540, 1620–6000, ≥ 18000) of sheep and goats.

To quantify the extent of genetic distance among *T. gondii* isolates from sheep and goats of Ethiopia, and evaluate their position towards reference strains from different continents, Neighbor-joining trees were reconstructed from the genetic distances among individual isolates using Populations 1.2.30 (1999, Olivier Langella, CNRS UPR9034, http://bioinformatics.org/~tryphon/populations/) and the Cavalli-Sforza and Edwards [23] (1967) chord-distance estimator. Unrooted trees were obtained with MEGA 6.06 software. The 95% confidence interval and a significance level of α = 0.05 were used.

### Ethical issues

This research project was approved by the animal ethical committee of the College of Veterinary Medicine and Agriculture, Addis Ababa University. All efforts were made to minimize animal suffering during the course of the study.

## Results

### Isolation of *T. gondii*

Viable *T. gondii* strains were isolated from 27 (57.45%) sheep and 20 (45.45%) goats. The sheep isolates were designated as TgSpEt 1 to 27 and the goat isolates were designated as TgGtEt 1 to 20 (see Additional file 2: Table S1 and Table S2). In general, isolation rate increased with titer of sheep and goats (*P* = 0.0001).

Additionally, viable *T. gondii* was isolated from 4 of 14 seronegative hearts (from 2 of 10 sheep and 2 of 4 goat samples. Each positive sample gave cyst positive result on one of the five mice). None of these cyst positive mice were DAT positive.

### Virulence

Two sheep isolates were virulent for mice. Isolate TgSpEt21 killed one mouse on 19 days post inoculation (dpi) and isolate TgSpEt19 killed 4 mice between 23–24 dpi. Additionally, one isolate from goat # 384 (designated as TgGtEt17) from East Shewa was mouse virulent as it killed one mouse on 27 dpi. Moreover, one sheep isolate (TgSpEt4) and two goat isolates (TgGtEt2 and TgGtEt11) each killed one mouse on day 44, 48 and 47, respectively (see Additional file 2: Table S1 and Table S2). One mouse inoculated with the sample of sheep # 539 was severely ill between 47–60 dpi, with signs of inappetance, dullness, weight loss, rough hair coat, paralysis, arched back and tachychardia.

### Genotyping

**Figure 1:**
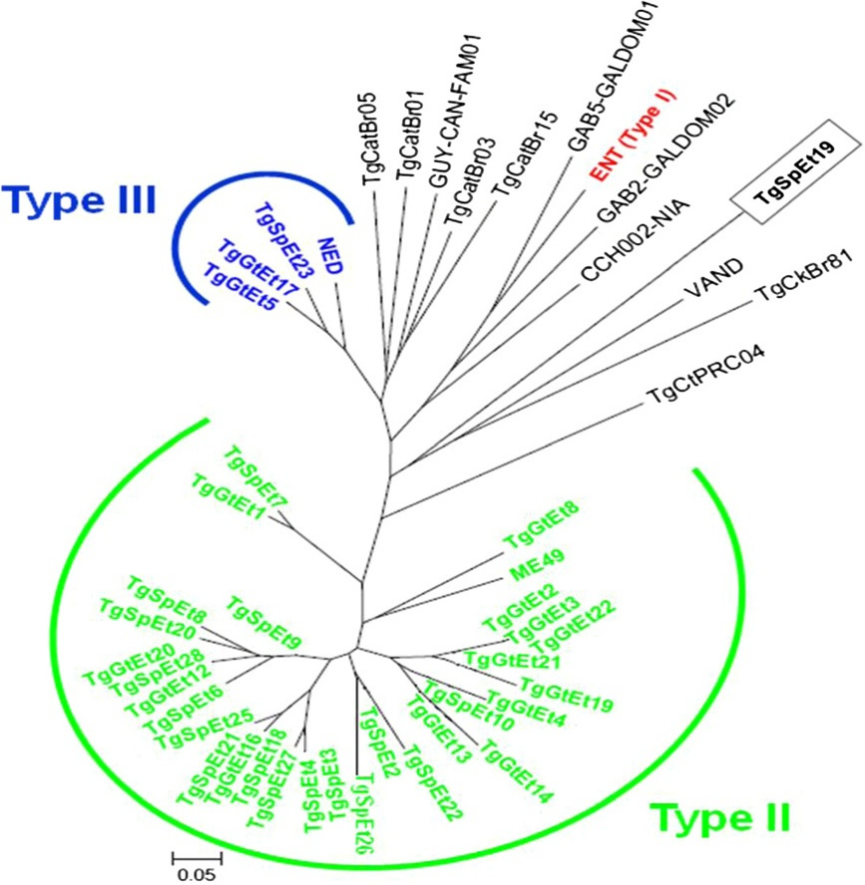
**Neighbor-joining analysis of 33** ***Toxoplasma gondii*** **strains isolated from sheep and goats (TgSp and TgGt) from Ethiopia and 14 reference strains with 15 microsatellite markers.** Color-coding indicates the three major clonal types: type I (red), type II (green), and type III (blue). The atypical isolate from Ethiopia, TgSpEt19, is highlighted by a black box.

In total, genotyping data with 15 MS markers were obtained from *T. gondii* isolates of 33 small ruminants of Central Ethiopia (18 sheep and 15 goats) and were compared to those obtained from 14 reference strains collected in America, Africa, Asia, and Europe (Table 1). The Neighbor-joining analysis of these 47 strains is displayed as an unrooted tree in the Figure 1. Out of the 33 strains from Ethiopia, 32 (97%) were clustered in only two groups: type II and type III (Figure 1). Type II was the most dominant being observed in 29 (87.87%) of the isolates followed by Type III (3/33, 9.09%). All isolates from West Shewa Zone were Type II. The allelic combination of the isolate TgSpEt19 was atypical and characterized by several unique alleles absent from the other reference strains (Table 1 and Figure 1). This isolate was found on a separate long branch in the Neighbor-joining tree and was clearly divergent from the type I, II, and III strains and did not group with the reference African strains (Figure 1).

**Table 1:** **Genotyping results of** ***T. gondii*** **DNA with 15 microsatellite markers from 33 isolates collected in sheep and goats of Central Ethiopia and from 14 reference strains collected in America, Africa, Asia, and Europe**

				Microsatellite markers ^1^														
Isolate	Type	Origin	Host	***TUB2***	***W35***	***TgM-A***	***B18***	***B17***	***M33***	***IV.1***	***XI.1***	***M48***	***M102***	***N60***	***N82***	***AA***	***N61***	***N83***
TgSpEt2	II	**W.Shewa**	Sheep	289	242	207	158	336	169	274	356	NA	176	NA	NA	NA	NA	NA
TgSpEt3	II	**W.Shewa**	Sheep	289	242	207	158	336	169	274	356	211	176	140	123	259	091	308
TgSpEt4	II	**W.Shewa**	Sheep	289	242	207	158	336	169	274	356	211	176	140	123	259	091	308
TgGtEt1	II	**W.Shewa**	Goat	289	242	207	158	336	169	274	356	225	172	142	117	259	093	308
TgGtEt2	II	**W.Shewa**	Goat	289	242	207	158	336	169	274	356	235	176	138	115	279	099	312
TgGtEt3	II	**W.Shewa**	Goat	289	242	207	158	336	169	274	356	235	176	138	115	279	099	312
TgGtEt5	III	**E.Shewa**	Goat	289	242	205	160	336	165	278	356	213	190	147	113	267	089	312
TgGtEt4	II	**W.Shewa**	Goat	289	242	207	158	336	169	274	356	237	176	140	113	273	093	312
TgSpEt6	II	**W.Shewa**	Sheep	289	242	207	158	336	169	274	356	213	176	140	115	265	117	308
TgGtEt8	II	**E.Shewa**	Goat	289	242	207	158	336	169	274	356	211	178	142	109	285	085	310
TgGtEt21^2^	II	**E.Shewa**	Goat	NA	242	NA	158	336	NA	NA	356	NA	NA	NA	NA	NA	NA	312
TgGtEt22^2^	II	**E.Shewa**	Goat	NA	242	NA	158	NA	NA	274	NA	NA	NA	NA	NA	279	NA	312
TgGtEt12	II	**E.Shewa**	Goat	289	242	207	158	336	169	274	356	213	176	140	115	261	089	308
TgSpEt10	II	**E.Shewa**	Sheep	289	NA	207	158	336	169	274	NA	NA	NA	NA	NA	NA	NA	312
TgSpEt28^2^	II	**E.Shewa**	Sheep	289	242	207	158	336	169	274	356	213	176	140	115	261	089	308
TgGtEt14	II	**E.Shewa**	Goat	289	242	207	158	336	169	274	356	233	174	140	113	269	097	312
TgGtEt13	II	**E.Shewa**	Goat	289	242	207	158	336	169	274	NA	NA	NA	140	113	NA	NA	NA
TgSpEt19	Atypical	**E.Shewa**	Sheep	289	240	201	156	340	163	272	360	209	218	147	127	283	099	342
TgGtEt17	III	**E.Shewa**	Goat	289	242	205	160	336	165	278	356	213	190	147	113	267	089	312
TgSpEt18	II	**E.Shewa**	Sheep	289	242	207	158	336	169	274	356	211	176	140	109	267	091	308
TgGtEt16	II	**E.Shewa**	Goat	289	242	207	158	336	169	274	356	211	176	140	109	267	091	308
TgSpEt22	II	**E.Shewa**	Sheep	289	242	207	158	336	169	274	356	219	176	140	111	267	097	310
TgSpEt20	II	**E.Shewa**	Sheep	289	242	207	158	336	169	274	356	213	178	140	115	261	091	308
TgSpEt21	II	**E.Shewa**	Sheep	289	242	207	158	336	169	274	356	211	176	140	109	267	091	308
TgGtEt19	II	**E.Shewa**	Goat	289	242	207	158	336	169	274	356	215	176	138	117	267	099	312
TgSpEt23	III	**E.Shewa**	Sheep	289	242	205	160	336	165	278	356	213	190	147	111	271	089	312
TgSpEt25	II	**E.Shewa**	Sheep	289	242	207	158	336	169	274	356	211	176	140	121	267	091	308
TgSpEt26	II	**E.Shewa**	Sheep	289	242	207	158	336	169	274	356	239	176	138	115	281	097	310
TgSpEt27	II	**E.Shewa**	Sheep	289	242	207	158	336	169	274	356	213	176	140	123	259	091	308
TgGtEt20	II	**E.Shewa**	Goat	289	242	207	158	336	169	274	356	213	176	140	115	261	089	308
TgSpEt7	II	**W.Shewa**	Sheep	289	242	207	158	336	169	274	356	225	172	142	117	261	093	308
TgSpEt8	II	**W.Shewa**	Sheep	289	242	207	158	336	169	274	356	213	178	140	115	265	089	308
TgSpEt9	II	**W.Shewa**	Sheep	289	242	207	158	336	169	274	356	NA	NA	140	115	NA	NA	316
GAB5-GAL-DOM01	Africa 1	**Gabon**	Chicken	291	248	205	160	342	165	274	354	231	166	149	111	277	087	306
CCH002-NIA	Africa 2	**Senegal**	Human	289	248	205	160	336	165	274	354	225	166	145	111	273	089	308
GAB2-GAL-DOM02	Africa 3	**Gabon**	Human	291	242	207	160	342	165	278	354	223	166	142	111	277	097	310
ENT	I	**France**	Human	291	248	209	160	342	169	274	358	209	166	145	119	267	087	306
ME49	II	**USA**	Sheep	289	242	207	158	336	169	274	356	215	174	142	111	265	091	310
NED	III	**France**	Human	289	242	205	160	336	165	278	356	209	190	147	111	267	091	312
VAND	Atypical	**French Guiana**	Human	291	242	203	162	344	167	276	356	217	170	142	113	277	091	308
GUY-CAN-FAM01	Caribbean 1	**French Guiana**	Dog	291	242	205	162	342	165	278	356	213	164	142	109	265	087	312
TgCtPRC04	Chinese 1	**China**	Cat	293	242	211	160	336	169	274	354	215	172	145	123	281	093	308
TgCatBr01	BR II	**Brazil**	Cat	289	242	205	160	342	165	278	358	233	164	147	111	316	089	308
TgCatBr03	BR III	**Brazil**	Cat	289	242	205	160	348	165	278	356	213	190	142	111	263	113	312
TgCatBr05	Atypical	**Brazil**	Cat	291	242	205	160	362	165	278	356	237	174	140	111	265	089	314
TgCatBr15	Atypical	**Brazil**	Cat	289	242	205	162	344	165	278	358	225	164	142	111	263	105	312
TgCkBr81	BR IV	**Brazil**	Chicken	291	246	207	162	362	169	272	358	217	164	142	105	322	081	314

W. Shewa = West Shewa Zone, E. Shewa = East Shewa Zone.

^1^NA, not amplified, ^2^ cyst negative but nPCR positive.

The three type III isolates were recovered from two goats and one sheep of East Shewa Zone. The atypical genotype was identified from a sheep of East Shewa Zone (Adea district). Only Type II genotypes were detected from West Shewa. Out of the three Type III isolates, one from goat # 384 killed one mouse on day 27 and another one from sheep # 539 made mice seriously ill from day 47–60 with debilitation and weight loss. The other Type III isolate was asymptomatic in mice. One Type II strain from East Shewa from sheep # 474 designated asTgSpEt21 was virulent for mouse.

Significant difference between DAT titer of sheep and goats in Ethiopia and genotypes (*P* = 0.027) as well as severity of disease in *T. gondii* infected mice and genotypes (*P* = 0.003) were detected with the Fisher’s exact test.

## Discussion

### Bioassay

The ingestion of undercooked mutton was identified as a risk for acquiring toxoplasmosis in pregnant women in Europe [24]. Considering the increasing trend and habit of eating raw or undercooked meat and offal from small ruminants in Ethiopia, the high rate of isolation of viable tissue cysts is of great public health significance. It is a reflection of the high degree of contamination of the environment by oocysts of cats and the outdoor management system of animals [1].

In the present study, viable *T. gondii* parasites were also isolated from 2 of 10 and 2 of 4 seronegative goats and sheep, respectively. The result might be related to the 1:40 cut-off used; viable *T. gondii* has been isolated from sheep and pigs in France with a low DAT titer (<1:16).

It appears that some of the inoculated mice in the present study failed to seroconvert even after developing brain cysts (Additional file 2: Table S1 and Table S2). It was also felt that the threshold DAT titer indicative of *T. gondii* infection in mice might be lower than 1:40. Isolation of viable *T. gondii* from DAT and dye test seronegative mice has been reported earlier [25, 26]. Some mice don’t seroconvert even at 8–10 weeks [27].

The present study indicates that 93.6% (44/47) of *T. gondii* strains circulating in the study area are non-virulent for mice. Two of the 27 *T. gondii* isolates from sheep and 1 of the 20 isolates from goats were virulent, since they killed mice between 19–27 dpi [19]. This is lower compared to a report from Brazil where, 9 of the 16 *T. gondii* isolates from sheep [28] and 10 of the 12 *T. gondii* isolates from goats [29] were mouse-virulent. The present mouse virulence detected was largely in agreement with *T. gondii* isolates from sheep in Europe and the United States where none of the 8 isolates from 30 seropositive adult ewes from France [30], and 52 of 53 isolates from lambs from the United States [31] were non-virulent for mice. Seropositive mice which died during the course of follow-up might be linked to the larger dose of bradyzoites received during i.p injection or to the strain of the parasite. Previous studies showed that the virulence of *T. gondii* in mice is related to the dose, route, stage of the parasite, genotype of the parasite, and strain of mice [32, 33].

### Genotyping

Most of the *T. gondii* isolates in the present study belonged to the type II clonal lineage (87.88%). The Type II clonal lineage is the most common lineage in Europe and North America; the Type III clonal lineage is found occasionally worldwide [5, 7, 15]. The dominance of Type II might be due to its fitness and enhanced ability to outcompete other genotypes (reviewed in Robert-Gangneux and Dardé, [3] as well as by its ability to form high numbers of cysts [4, 34]. The Type II genetic lineage has been shown to be the dominant lineage in opportunistic infections in immunocompromised people and in congenital toxoplasmosis in Europe [8, 35], and was found in 73% of cases of ocular disease in France [36]. In contrast, the absence of Type II and overabundance of atypical genotypes are characteristic of *T. gondii* strains from continental Brazil [33].

All isolates from West Shewa were identified as Type II while that of East Shewa additionally contained Type III and atypical lineages. The higher genetic diversity of *T. gondii* detected in East Shewa could be ascribed to diverse agro-ecologies in this area (altitude ranging from 953 to 3400 masl). Ethiopia served as a historical entry point of many livestock populations from Asia, thousands years ago [37]. The dominance of clonal lineage (Type II) in the present study is in accordance with the long history of breeding and domestication of companion and agricultural animals in Ethiopia [37] by mankind and consequent modification of natural biotopes that usually results in a loss of diversity [38, 39]. This in turn decreases the probability of genetic recombination and emergence of new genotype in cats through simultaneously feeding on preys harbouring different genotypes. On the other hand, the presence of a highly divergent atypical genotype in East Shewa suggests the existence of an African gene pool with endemic *T. gondii* alleles in Ethiopia different from Type II. Our results in Central Ethiopia and other study in Ethiopia [15] showed that Type II strains dominate the genetic diversity in domestic animals from this part of Africa. Atypical genotypes with a different gene pool appear to be rare in anthropized environments of Ethiopia but may be more abundant in the wild where Type II strains didn’t have the opportunity to penetrate yet.

The Type II and III genetic lineages (which constitute about 97% of the isolates of the present study) were previously also reported from feral cats of Addis Ababa. This shows that these are the most common and widespread genetic lineages of *T. gondii* in Central Ethiopia. The atypical genotype from the strain TgSpEt19 isolated in a sheep from Adea district (East Shewa Zone) comprises alleles for the markers *N61*, *B18*, *N83*, *XI-I*, *N82*, *TgM-A*, *IV-I*, *B17*, *N60* and *AA* that are markedly different from allelic composition of Type II and III strains isolated in this study (Table 1). It might perhaps be unique among isolates from other countries ever genotyped using the multiplex-PCR microsatellite method (Ajzenberg, D., personal communication). Thus, further large-scale investigation on different hosts and geographical areas in Ethiopia might reveal more atypical genotypes significantly divergent from the conventional Type I, II and III lineages. Identification of one atypical strain virulent in mice from Arsi-Bale sheep breed of Adea district is of considerable clinical and epidemiological importance as atypical strains are considered to be more pathogenic to humans than type II or III strains. For example, in immunocompetent patients, severe toxoplasmosis with multi-organ failure has been linked to atypical strains acquired from the Amazonian rainforest [40]. Similarly, severe cases of congenital toxoplasmosis in France [7, 41] and Suriname [42] and abortion in sheep in Uruguay [5, 43] were observed in relation to infection with atypical genotypes. Severe or lethal infections in immunocompetent subjects, with pneumonitis, myocarditis, meningoencephalitis, or polymyositis were also reported due to atypical genotypes [7]. Outdoor access of domestic cats, abundance of feral cats and wild felids coupled with widely practiced extensive management of animals in Ethiopia might favour high rate of transmission of toxoplasmosis with occasional possibility of cats being infected with two different genotypes followed by sexual recombination and emergence of atypical genotypes [15, 33]. Supporting this reasoning, Dubey et al. [15] attributed the isolation of different genotypes from tissues and feces of two feral cats (out of 27 isolates) of Addis Ababa to re-infection or mixed strain infection.

## Conclusions

The present findings indicate a high rate of isolation of *T. gondii* from seropositve Ethiopian small ruminants slaughtered for human consumption. The present study also revealed three genetic lineages (Type II, III and atypical) of *T. gondii*. This is the first report of isolation and molecular characterization of *T. gondii* infection in mice experimentally infected with heart homogenates of seropositive sheep and goats in Ethiopia.

## Electronic supplementary material

Additional file 1: **Microsatellite markers and PCR primers used for the multiplex PCR assay.** (DOC 40 KB)

Additional file 2: Table S1: Isolation of *T. gondii* from seropositive sheep of East and West Shewa Zones, Central Ethiopia. **Table S2.** Isolation of *T. gondii* from seropositive goats of East and West Shewa Zones, Central Ethiopia. (DOC 148 KB)
