# Supplementary material for: Isolation and genotyping of viable Toxoplasma gondii from sheep and goats in Ethiopia destined for human consumption
Source: Parasit Vectors. 2014 Sep 4;7:425. doi: 10.1186/1756-3305-7-425 (PMC4161867; doi:10.1186/1756-3305-7-425)
Supplement: Supplementary file 1 — Additional file 1: Microsatellite markers and PCR primers used for the multiplex PCR assay. (DOC 40 KB) [file 13071_2014_1583_MOESM1_ESM.doc]

Additional file 1. Microsatellite markers and PCR primers used for the multiplex PCR assay

| Marker | Chromosome (position)^a^ | Repeat motif(s) | Primer sequence^b^ | Size range  (bp) |
| --- | --- | --- | --- | --- |
| TUB2 | IX (974608 to 974896) | [TG/AC]n | (F) 5’ 6-FAM-GTCCGGGTGTTCCTACAAAA 3’  (R) 5’ TTGGCCAAAGACGAAGTTGT 3’ | 287–291 |
| W35 | II (633241 to 633482) | [TC/AG]n, [TG/AC]n | (F) 5’ HEX-GGTTCACTGGATCTTCTCCAA 3’  (R) 5’ AATGAACGTCGCTTGTTTCC 3’ | 242–248 |
| TgM-A | X (4824879 to 4825083) | [TG/AC]n | (F) 5’HEX-GGCGTCGACATGAGTTTCTC 3’  (R) 5’ TGGGCATGTAAATGTAGAGATG 3’ | 203–211 |
| B18 | VIIa (2921536 to 2921693) | [TG/AC]n | (F) 5’ 6-FAM-TGGTCTTCACCCTTTCATCC 3’  (R) 5’ AGGGATAAGTTTCTTCACAACGA 3’ | 156–170 |
| B17 | XII (6474746 to 6475079) | [TC/AG]n | (F) 5’ HEX-AACAGACACCCGATGCCTAC 3’  (R) 5’ GGCAACAGGAGGTAGAGGAG 3’ | 334–366 |
| M33 | IV (672591 to 672760) | [TC/AG]n | (F) 5’ 6-FAM- TACGCTTCGCATTGTACCAG 3’  (R) 5’ TCTTTTCTCCCCTTCGCTCT 3’ | 165–173 |
| IV.1 | IV (742419 to 742693) | [TG/AC]n | (F) 5’ HEX-GAAGTTCGGCCTGTTCCTC 3’  (R) 5’ TCTGCCTGGAAAAGGAAAGA 3’ | 272–282 |
| XI.1 | XI (189702 to 190058) | [TG/AC]n | (F) 5’ 6-FAM-GCGTGTGACGAGTTCTGAAA 3’  (R) 5’ AAGTCCCCTGAAAAGCCAAT 3’ | 354–362 |
| M48 | Ia (332951 to 333166) | [TA/AT]n | (F) 5’ 6-FAM-AACATGTCGCGTAAGATTCG 3’  (R) 5’ CTCTTCACTGAGCGCCTTTC 3’ | 209–243 |
| M102 | VIIa (3093491 to 3093664) | [TA/AT]n | (F) 5’NED-CAGTCCAGGCATACCTCACC 3’  (R) 5’ CAATCCCAAAATCCCAAACC 3’ | 164–196 |
| N60 | Ib (1766079 to 1766221) | [TA/AT]n | (F) 5’ NED-GAATCGTCGAGGTGCTATCC 3’  (R) 5’ AACGGTTGACCTGTGGCGAGT 3’ | 132–157 |
| N82 | XII (1621472 to 1621585) | [TA/AT]n | (F) 5’HEX-TGCGTGCTTGTCAGAGTTC 3’  (R) 5’ GCGTCCTTGACATGCACAT 3’ | 105–145 |
| AA | VIII (5836880 to 5837144) | [TA/AT]n | (F) 5’ NED-GATGTCCGGTCAATTTTGCT 3’  (R) 5’ GACGGGAAGGACAGAAACAC 3’ | 251–332 |
| N61 | VIIb (4217145 to 4217238) | [TA/AT]n | (F) 5’ 6-FAM-ATCGGCGGTGGTTGTAGAT 3’ (R) 5’ CCTGATGTTGATGTAAGGATGC 3’ | 79-123 |
| N83 | X (1772898 to 1773209) | [TA/AT]n | (F) 5’ 6-FAM-ATGGGTGAACAGCGTAGACA3’  (R) 5’ GCAGGACGAAGAGGATGAGA 3’ | 306–338 |

*a* Based on the position within each chromosome of strain ME49 as given in the Toxo DB website (http://toxodb.org.

*b* (F), forward primer; (R), reverse primer. In each pair of primers, the forward primer was labeled at the 5’ end with fluorescein: 6-carboxyfluorescein (6-FAM) for MS *TUB2*, *XI.1*, *B18*, *N83*, *N61*, *M33*, and *M48*, hexachlorofluorescein (HEX) for MS *TgM-A*, *B17*, *N82*, *W35*, and *IV.1*, and 2’,7’,8’-benzo-5’-fluoro-2’,4,7-trichloro-5-carboxyfluorescein (NED) for MS *AA*, *N60*, and *M10*2.
