# Supplementary material for: Isolation and genotyping of viable Toxoplasma gondii from sheep and goats in Ethiopia destined for human consumption
Source: Parasit Vectors. 2014 Sep 4;7:425. doi: 10.1186/1756-3305-7-425 (PMC4161867; doi:10.1186/1756-3305-7-425)
Supplement: Supplementary file 2 — Additional file 2: Table S1: Isolation of T. gondii from seropositive sheep of East and West Shewa Zones, Central Ethiopia. Table S2. Isolation of T. gondii from seropositive goats of East and West Shewa Zones, Central Ethiopia. (DOC 148 KB) [file 13071_2014_1583_MOESM2_ESM.doc]

Additional file 2: Table S1 Isolation of *T. gondii* from seropositive sheep of East and West Shewa Zones, Central Ethiopia

| Origin | Animal Id | Reciprocal MAT titer of seropositive sheep | Bioassay in mice | | | | Isolate designation |
| --- | --- | --- | --- | --- | --- | --- | --- |
|  |  |  | Mice with cysts/exam.(n) | Seropositive mice/mice examined (n) | Mice died/infected | Days of mice death PI (mice no.) |  |
| West Shewa | 68 | 54000 | 1/5 | 0/5 | 0/5 | Survived | TgSpEt1 |
|  | 72 | ≥162000 | 0/5 | 0/5 | 0/5 | Survived | - |
|  | 94 | ≥162000 | 3/3 | 3/3 | 2/5 | 2 (2) | TgSpEt2 |
|  | 108 | 54000 | 1/3 | 3/4 | 1/5 | 2 (1) | TgSpEt3 |
|  | 110 | ≥162000 | 1/1 | 1/1 | 3/5 | **44 (1)**, 2 (2). 3 (1) | TgSpEt4 |
|  | 112 | ≥162000 | 0/3 | 0/3 | 2/5 | 2 (2) | - |
|  | 167 | ≤60 | 0/5 | 0/5 | 0/5 | Survived | - |
|  | 172 | ≤60 | 0/1 | 0/1 | 4/5 | 2 (4) | - |
|  | 173 | ≤60 | 0/5 | 0/5 | 0/5 | Survived | - |
|  | 175 | ≤60 | 0/5 | 0/5 | 0/5 | Survived | - |
|  | 198 | 1620 | 0/5 | 1/5 | 0/5 | Survived | - |
|  | 202 | 1620 | 0/5 | 0/5 | 0/5 | Survived | - |
|  | 203 | ≤60 | 1/5 | 1/5 | 0/5 | Survived | TgSpEt5 |
|  | 204 | ≥162000 | 5/5 | 5/5 | 0/5 | Survived | TgSpEt6 |
|  | 324 | ≤60 | 0/5 | 0/5 | 0/5 | Survived | - |
|  | 599 | ≥162000 | 2/5 | 3/5 | 0/5 | Survived | TgSpEt7 |
|  | 603 | ≥162000 | 5/5 | 5/5 | 0/5 | Survived | TgSpEt8 |
|  | 604 | ≥162000 | 4/6 | 6/6 | 0/6 | Survived | TgSpEt9 |
| East Shewa | A1 | ≤60 | 0/3 | 0/3 | 2/5 | 2 (2) | - |
|  | 81 | 54000 | 0/5 | 0/5 | 0/5 | Survived | - |
|  | 251 | 180 | 0/5 | 0/5 | 0/5 | Survived | - |
|  | 256 | 54000 | 0/5 | 0/5 | 0/5 | Survived | - |
|  | 257 | 54000 | 3/5 | 3/5 | 0/5 | Survived | TgSpEt10 |
|  | 258 | 1620 | 2/5 | 2/5 | 0/5 | Survived | TgSpEt11 |

Additional file 2: Table S1 Continued…

| Origin | Animal Id | Reciprocal MAT titer of seropositive sheep | Bioassay in mice | | | | Isolate designation |
| --- | --- | --- | --- | --- | --- | --- | --- |
|  |  |  | Mice with cysts/exam.(n) | Seropositive mice/mice examined (n) | Mice died/infected | Days of mice death PI (mice no.) |  |
| East Shewa | 259 | 54000 | 0/5 | 0/5 | 0/5 | Survived | - |
|  | 260 | 1620 | 1/4 | 0/4 | 1/5 | 2 (1) | TgSpEt12 |
|  | 261 | 54000 | 1/5 | 5/5 | 0/5 | Survived | TgSpEt13 |
|  | 357 | 6000 | 1/5 | 0/5 | 0/5 | Survived | TgSpEt14 |
|  | 360 | 18000 | 0/5 | 0/5 | 0/5 | Survived | - |
|  | 362 | 1620 | 2/5 | 4/5 | 0/5 | Survived | TgSpEt15 |
|  | 410 | ≤60 | 1/2 | 0/2 | 3/5 | 2 (3) | TgSpEt16 |
|  | 426 | 6000 | 0/5 | 0/5 | 0/5 | Survived | - |
|  | 430 | 18000 | 2/5 | 4/5 | 0/5 | Survived | TgSpEt17 |
|  | 447 | 18000 | 5/5 | 5/5 | 0/5 | Survived | TgSpEt18 |
|  | 448 | 54000 | 3/5 | 5/5 | 4/5 | **23 (1),** **^24 (3)^** | TgSpEt19 |
|  | 449 | ≤60 | 0/5 | 0/5 | 0/5 | Survived | - |
|  | 470 | ≤60 | 0/4 | 0/4 | 1/5 | 2 (1) | - |
|  | 471 | 54000 | 1/1 | 1/1 | 4/5 | 2 (4) | TgSpEt20 |
|  | 474 | 180 | 1/2 | 0/2 | 3/5 | 1(1), 2(1), **^19(1)^** | TgSpEt21 |
|  | 501 | ≥162000 | 1/1 | 1/1 | 4/5 | 2(4) | TgSpEt22 |
|  | 536 | 6000 | 0/5 | 0/5 | 0/5 | Survived | - |
|  | 537 | 180 | 0/5 | 0/5 | 0/5 | Survived | - |
|  | 539 | 6000 | 4/5 | 5/5 | 0/5 | Survived,1 sick | TgSpEt23 |
|  | 540 | 6000 | 2/4 | 2/4 | 1/5 | 3(1) | TgSpEt24 |
|  | 545 | 6000 | 5/5 | 5/5 | 0/5 | Survived | TgSpEt25 |
|  | 551 | 54000 | 5/5 | 4/5 | 0/5 | Survived | TgSpEt26 |
|  | 552 | ≥162000 | 1/2 | 1/2 | 3/5 | 3(3) | TgSpEt27 |

Additional file 2: Table S2 Isolation of *T. gondii* from seropositive goats of East and West Shewa Zones, Central Ethiopia

| Origin | Animal ID | Reciprocal MAT titer of seropositive goats | Bioassay in mice | | | | Isolate designation |
| --- | --- | --- | --- | --- | --- | --- | --- |
|  |  |  | Mice with cysts/mice  examined (n) | Seropositive mice/mice  examined (n) | Mice died/  infected | Days of mice death PI  (no. of mice dead) |  |
| West Shewa | 142 | 1620 | 4/5 | 4/5 | 0/5 | Survived | TgGtEt 1 |
|  | 176 | 54000 | 5/5 | 5/5 | 1/6 | **48 (1)** | TgGtEt 2 |
|  | 182 | ≤60 | 1/5 | 1/5 | 0/5 | Survived | TgGtEt 3 |
|  | 186 | ≤60 | 0/4 | 1/4 | 3/5 | 2 (1) | - |
|  | 190 | 180 | 0/5 | 0/5 | 0/5 | Survived | - |
|  | 191 | 540 | 0/5 | 0/5 | 0/5 | Survived | - |
|  | 192 | ≥162000 | 5/5 | 5/5 | 0/5 | Survived | TgGtEt 4 |
|  | 600 | ≥162000 | 0/5 | 1/5 | 0/5 | Survived | - |
| East Shewa | A2 | 6000 | 5/5 | 5/5 | 0/5 | Survived | TgGtEt 5 |
|  | A3 | ≤60 | 0/5 | 0/5 | 0/5 | Survived | - |
|  | A6 | 1620 | 0/5 | 0/5 | 0/5 | Survived | - |
|  | A14 | ≤60 | 1/5 | 0/5 | 0/5 | Survived | TgGtEt 6 |
|  | A32 | 540 | 0/5 | 1/5 | 0/5 | Survived | - |
|  | 91 | 54000 | 0/5 | 0/5 | 0/5 | Survived | - |
|  | 132 | 1620 | 0/5 | 0/5 | 0/5 | Survived | - |
|  | 133 | ≤60 | 0/4 | 0/4 | 1/5 | 2 (1) | - |
|  | 218 | ≤60 | 1/5 | 0/5 | 0/5 | Survived | TgGtEt 7 |
|  | 220 | 540 | 0/5 | 0/5 | 0/5 | Survived | - |
|  | 221 | 180 | 0/4 | 0/4 | 1/5 | 2 (1) | - |
|  | 222 | 1620 | 0/5 | 0/5 | 0/5 | Survived | - |
|  | 223 | 1620 | 0/4 | 0/4 | 1/5 | 2 (1) | - |
|  | 224 | ≥162000 | 5/5 | 5/5 | 0/5 | Survived | TgGtEt 8 |

Additional file 2: Table S2 Continued….

| Origin | Animal ID | Reciprocal MAT titer of seropositive goats | Bioassay in mice | | | | Isolate designation |
| --- | --- | --- | --- | --- | --- | --- | --- |
|  |  |  | Mice with cysts/mice  examined (n) | Seropositive mice/mice  examined (n) | Mice died/  infected | Days of mice death PI  (no. of mice dead) |  |
| East Shewa | 225 | 180 | 0/3 | 0/3 | 2/5 | 2(1),3(1) | - |
|  | 230 | 54000 | 0/5 | 0/5 | 0/5 | Survived | - |
|  | 231 | 18000 | 0/5 | 0/5 | 0/5 | Survived | - |
|  | 234 | 180 | 0/5 | 0/5 | 0/5 | survived | - |
|  | 236 | 1620 | 0/3 | 0/3 | 2/5 | 2(2) | - |
|  | 237 | 18000 | 1/5 | 1/5 | 0/5 | Survived | TgGtEt 9 |
|  | 239 | 1620 | 2/4 | 0/4 | 1/5 | 2(1) | TgGtEt 10 |
|  | 240 | 180 | 1/6 | 0/6 | 1/6 | **47 (1)** | TgGtEt 11 |
|  | 250 | 54000 | 2/5 | 0/5 | 0/5 | Survived | TgGtEt 12 |
|  | 312 | ≤60 | 1/3 | 1/3 | 2/5 | 2(2) | TgGtEt 13 |
|  | 313 | ≤60 | 0/5 | 0/5 | 1/5 | 1 (1) | - |
|  | 315 | ≤60 | 0/5 | 0/5 | 0/5 | Survived | - |
|  | 321 | 18000 | 5/5 | 1/5 | 0/5 | Survived | TgGtEt 14 |
|  | 349 | ≤60 | 1/5 | 0/5 | 0/5 | Survived | TgGtEt 15 |
|  | 373 | ≤60 | 2/3 | 3/3 | 2/5 | 1(1), 2(1) | TgGtEt 16 |
|  | 384 | ≤60 | 2/3 | 2/2 | 3/5 | ^3(2)^, **^27 (1)^** | TgGtEt 17 |
|  | 396 | ≤60 | 2/5 | 0/5 | 0/5 | Survived | TgGtEt 18 |
|  | 514 | ≤60 | 0/2 | 2/2 | 3/5 | 2(3) | - |
|  | 521 | 54000 | 3/4 | 4/4 | 1/5 | 3 (1) | TgGtEt 19 |
|  | 562 | ≥162000 | 4/7 | 6/7 | 0/7 | Survived | TgGtEt 20 |
|  | 573 | ≤60 | 0/5 | 0/5 | 0/5 | Survived | - |
|  | 578 | ≤60 | 0/5 | 1/5 | 0/5 | Survived | - |
